# Evolving complexities of infant HIV diagnosis within Prevention of Mother-to-Child Transmission programs

**DOI:** 10.12688/f1000research.19637.1

**Published:** 2019-09-13

**Authors:** Ahmad Haeri Mazanderani, Gayle G. Sherman

**Affiliations:** 1 Centre for HIV & STIs, National Institute for Communicable Diseases, National Health Laboratory Service, Johannesburg, South Africa; 2 Department of Medical Virology, Faculty of Health Sciences, University of Pretoria, Pretoria, South Africa; 3 Department of Paediatrics & Child Health, Faculty of Health Sciences, University of the Witwatersrand, Johannesburg, South Africa

**Keywords:** early infant diagnosis, prevention of mother to child transmission, antiretroviral therapy

## Abstract

Early diagnosis of HIV infection among infants and children is critical as prompt initiation of antiretroviral therapy prevents morbidity and death. Yet despite advances in the accuracy and availability of infant HIV diagnostic testing, there are increasing challenges with making an early definitive diagnosis. These challenges relate primarily to advances in prevention of mother-to-child transmission (PMTCT) of HIV. Although PMTCT programs have proven to be highly effective in reducing infant HIV infection, infants who are HIV-infected may achieve virological suppression and loss of detectability of HIV nucleic acid prior to diagnosis because of antiretroviral drug exposure. Hence, false-negative and indeterminate HIV polymerase chain reaction (PCR) results can occur, especially among high-risk infants given multi-drug prophylactic regimens. However, the infant HIV diagnostic landscape is also complicated by the inevitable decline in the positive predictive value of early infant diagnosis (EID) assays. As PMTCT programs successfully reduce the mother-to-child transmission rate, the proportion of false-positive EID results will increase. Consequently, false-negative and false-positive HIV PCR results are increasingly likely despite highly accurate diagnostic assays. The problem is compounded by the seemingly intractable prevalence of maternal HIV within some settings, resulting in a considerable absolute burden of HIV-infected infants despite a low mother-to-child transmission rate.

## Introduction

Early diagnosis of HIV infection among infants and young children is critical as prompt initiation of antiretroviral therapy (ART) markedly reduces morbidity and mortality ^1^. However, making an early definitive diagnosis of HIV is becoming increasingly difficult despite improvements in the accuracy and availability of infant diagnostic testing. Importantly, the challenges associated with early infant diagnosis (EID) relate directly to improvements in prevention of mother-to-child transmission (PMTCT) of HIV.

As a result of the passive transfer of maternal HIV antibodies to infants in the third trimester and the persistence of these antibodies during infancy and early childhood, HIV antibody tests used to diagnose HIV in older children and adults cannot be used for EID. Rather, detection of HIV antibodies in infants, as determined by enzyme-linked immunosorbent assays (ELISAs) and rapid diagnostic tests, indicates HIV exposure (that is, maternal infection) but not necessarily infant infection. Hence, to diagnose HIV infection in infants, nucleic acid tests, such as HIV polymerase chain reaction (PCR) assays, are used. These tests directly detect HIV DNA, RNA, or total nucleic acid and may be qualitative or quantitative, the latter referred to as HIV viral load assays.

Whereas the first nucleic acid tests for HIV diagnosis were beset with challenges, including suboptimal amplification of HIV subtype C and carry-over contamination, assay refinements and development of fully automated closed systems led to marked improvement in EID accuracy. An increasing number of commercial assays approved for *in vitro* diagnostic use are now available. These range from fully automated high-throughput real-time PCR closed systems, designed for centralized laboratories, to single-test point-of-care devices with vastly reduced analytical turnaround times. Yet despite these advances, there are increasing challenges with making an early definitive HIV diagnosis among infants and young children. In this review article, developments and successes in the field of PMTCT, including nucleic acid and antibody testing and their implication for pediatric HIV diagnosis, will be presented.

## Early infant diagnosis and antiretroviral drug exposure

EID not only is essential for clinical decision making (namely, timely identification of HIV-infected infants, thereby facilitating linkage to care and ART initiation) but also provides an opportunity to measure the effectiveness of PMTCT programs by documenting transmission rates. For example, routine laboratory data from South Africa’s National Health Laboratory Service demonstrated the successful reduction of early infant infection from more than 20% in 2004 to less than 2% by 2015 among HIV-exposed infants ^2, 3^. This reduction in mother-to-child transmission was achieved by increasing access to maternal treatment and infant prophylaxis regimens as well as lowering the threshold for maternal ART initiation. PMTCT prophylaxis, originally recommended only around the time of childbirth, has been progressively expanded to safeguard the pregnancy and postpartum period. The World Health Organization (WHO) currently recommends lifelong ART for all HIV-infected pregnant women regardless of CD4 count or clinical stage; this is referred to as WHO PMTCT Option B+. Hence, there is a growing population of women living with HIV who are initiated on suppressive ART regimens for months, if not years, prior to delivery. This in turn has altered the epidemiology of early infant HIV infection.

Mother-to-child transmission of HIV can arise from one of three routes: transplacentally (intrauterine infection), exposure to blood/secretions at time of delivery (intrapartum infection), or via breastmilk (postnatal infection). Importantly, the risk of infection from each transmission route is directly related to maternal viremia. Prior to the ART era, intrapartum infections were the predominant mode of transmission among formula-fed infants and accounted for up to 50% of all HIV infections among breastfed infants ^4^. Therefore, routine HIV PCR testing at 4 to 6 weeks of age has been the mainstay of EID testing as both intrauterine ** and intrapartum infections can be detected at a single time point which coincides with a routine immunization visit ( Table 1). However, as access to ART has increased, the proportion of viremic women at delivery has decreased. Consequently, intrapartum transmissions have disproportionately declined, thereby reversing the intrauterine-to-intrapartum transmission ratio to about 3:1, albeit within the context of an overall reduced mother-to-child transmission rate ^5^. This change in the epidemiology of infant infection is relevant as intrauterine infected infants have a more rapid disease onset and higher risk of mortality than those infected through other transmission routes ^6– 8^. Indeed, findings from South Africa have suggested that within a 6-week testing program up to 20% of intrauterine infected infants died or were lost to follow-up before 6 weeks of age ^9, 10^. This has prompted a revision of EID guidelines to support routine birth testing among all HIV-exposed infants followed by a second HIV PCR test at 6 weeks of age (to detect possible intrapartum infections among those infants who tested negative at birth) ^11^.

**Table 1.** Updated recommendations for HIV testing of infants and children.

HIV test	Previous recommendations for time of testing	Current recommendations for time of testing
HIV nucleic acid test	- 4 to 6 weeks of age - 6 weeks after cessation of breastfeeding If <18 months of age	- Birth - 6 or 10 weeks of age - 6 or 9 months of age - 3 months after cessation of breastfeeding If <18 months of age
HIV antibody test	- 9 months (HIV-exposed only) If positive, confirm with an HIV polymerase chain reaction (PCR) test - 18 months (HIV-exposed children only) If positive, confirm with a second antibody test - 6 weeks after cessation of breastfeeding If >18 months of age	- 18 months (all children) If positive and <24 months, confirm with an HIV PCR test - 3 months after cessation of breastfeeding If >18 months of age

South Africa introduced routine birth testing in 2015 and rapidly achieved a testing coverage of 95% within the first year of implementation ^12^. In order to detect intrapartum infections early, a second EID test was also included in the national infant testing guidelines. Importantly, HIV testing occurs within the context of PMTCT practices and considerable exposure to antiretroviral drugs. In addition to recommending antenatal maternal ART, which results in significant intrauterine ** antiretroviral exposure ^13^, WHO PMTCT Option B+ recommends that all HIV-exposed infants receive daily antiretroviral prophylaxis for 4 to 6 weeks regardless of feeding method ^14^. Studies suggest that infant prophylactic regimens can result in virological suppression and loss of detectability by PCR assays in HIV-infected infants ^15– 18^. For these reasons, guidelines from the US recommend that infants at high risk of HIV transmission undergo repeat nucleic acid testing 2 to 4 weeks after cessation of antiretroviral prophylaxis ^19^. Out of similar concern, South Africa’s testing guidelines recommend routine HIV PCR testing at 10 weeks post-delivery, instead of 6 weeks, for detection of intrapartum infections.

In addition to impacting on the sensitivity of EID screening, antiretroviral drug exposure can complicate confirmatory testing. Guidelines recommend that infants who test HIV PCR-positive be recalled as a matter of urgency to both confirm their HIV infection status and initiate ART at the same visit. However, in some circumstances, confirmatory testing is performed after prolonged exposure to antiretroviral prophylaxis and sometimes even after ART initiation. Unsurprisingly, the confirmatory HIV test may yield an indeterminate or even a negative result even when the infant is infected. Recalling the infant for testing to resolve the discordant HIV test results is further complicated by additional reductions in virus. Hence, the longer the intervals between testing, the higher the risk of viral suppression and discordant HIV test results. Among infants with discordant results who have already initiated ART, treatment interruption represents a last resort for making a definitive diagnosis provided that close monitoring and repeat HIV testing can be ensured.

## Infant feeding, postnatal transmission, and HIV testing during early childhood

Delayed HIV detection is also reported among infants exposed to postnatal antiretroviral prophylaxis during breastfeeding ^20^. Since 2010, the WHO has recommended ART interventions to prevent postnatal transmission of HIV and has subsequently updated guidelines to report that mothers living with HIV should breastfeed for at least 12 months and may continue breastfeeding for up to 24 months or longer whilst supported for ART adherence ^21^. However, there are concerns about maternal ART adherence among women initiated on treatment during pregnancy, and frequent viremic episodes are reported during the postpartum period ^22^. Hence, there is an increasing need for HIV testing services and postnatal surveillance within PMTCT programs, especially in light of the fact that postnatal transmission accounts for the majority of infant HIV infections within some high-burden settings ^23^.

As HIV infection can arise at any time during breastfeeding and duration of breastfeeding is variable, a programmatic approach to prompt postnatal infant diagnosis is challenging. The WHO recommends repeat HIV testing for all infants 3 months (previously 6 weeks) after breastfeeding cessation - if the infant/child is less than 18 months of age at weaning then PCR testing is recommended, whereas if the child is more than 18 months of age then antibody testing is recommended ^24^. However, as weaning does not necessarily coincide with a planned clinic visit, testing coverage at this time point has been poor. To address the deficit in postnatal testing, the WHO originally recommended a screening antibody test at 9 months of age for all HIV-exposed infants. Infants testing positive required a positive HIV PCR test to confirm infection. The rationale for using antibody testing was to optimize access whilst limiting the high costs of nucleic acid tests. With improved access to and reduced costs of virological tests in recent times, PCR has replaced antibody testing at 9 months of age ^25^.

A nationally representative sample of South African HIV-exposed infants demonstrated 4.3% (95% confidence interval 3.7–5%) cumulative mother-to-child transmission at 18 months of age in 2012/13, and 81% of all transmissions occurred by 6 months of age ^26^. Considering this and the delay caused by ART in detecting perinatal transmission and decreasing immunization coverage with age, South Africa has positioned a third HIV PCR test at the 6-month instead of the 9-month immunization visit ^27^.

In the pre-ART era, the majority of HIV-exposed uninfected infants had seroreverted (that is, lost maternal HIV antibodies) by 9 months of age, and some infants remained seropositive up until 18 months. Recent data from South Africa suggest that seroreversion rates at 9 months, using the same rapid test, decreased from 82 to 50% among HIV-exposed uninfected infants between 2005 and 2016 ^28, 29^. Delayed seroreversion beyond 18 months of age using ELISA testing is also reported, and detection of maternal antibodies up until 24 months of age was associated with maternal ART use and was thought to result from the transfer from healthier mothers to their unborn infants of higher concentrations of HIV antibodies that take longer to decay ^30^. Accordingly, a recommendation to confirm the HIV infection status of serologically positive HIV-exposed infants with a nucleic acid test between the ages of 18 and 24 months has been made to avoid a false-positive diagnosis with the consequence of lifelong ART ^27^.

Although routine HIV serology testing at 18 months of age has been standard of care for many years, identification of HIV exposure at the 18-month immunization visit has been problematic, leading to poor testing coverage ^31^. Increased risk of HIV acquisition among women throughout pregnancy and the postnatal period has also likely caused some HIV-exposed infants to be incorrectly classified as unexposed ^32^. To ensure that no case of vertical transmission goes undetected, high-prevalence settings like South Africa are recommending that all children undergo HIV antibody testing at 18 months of age regardless of documented HIV exposure status ^27^. However, universal 18-month HIV testing may pose a challenge among certain populations. For example, children testing positive at or after 18 months of age may be vaccine trial participants (for example, immunization with broadly neutralizing antibodies for prevention of vertical transmission) in whom a positive antibody test does not necessarily indicate HIV infection. Conversely, HIV-infected infants initiated on ART who achieve prolonged virological suppression can test HIV antibody–negative because of a lack of antigenic stimulation. Hence, if caregivers fail to disclose the status of children already initiated on ART or if health-care workers are unaware that HIV-infected patients can serorevert, false-negative results may arise which could negatively impact treatment adherence.

## HIV prevalence, early infant diagnosis positive predictive value, and indeterminacy

The reduced diagnostic sensitivity of HIV assays as a result of ART exposure is only part of the unfolding narrative of infant diagnosis. As PMTCT programs successfully reduce mother-to-child transmission, the positive predictive value of EID tests is expected to decline, resulting in an increasing proportion of false-positive results. For example, where the specificity of a test is 99.9% and the HIV prevalence in the tested population is 5%, the expected positive predictive value of the test is 98% (that is, 2% false-positive rate). However, when the same test is used in a setting with a 1% prevalence, the positive predictive value decreases to 91% (that is, 9% false-positive rate). To address this, the WHO has proposed using an indeterminate range, defined as viral copy equivalents too low to confer an accurate positive result, to improve the accuracy of all nucleic acid–based EID assays ^24, 33^.

Proposed indeterminate cutoff criteria are based on laboratory findings of poor positive predictive value and irreproducible positive results associated with higher cycle threshold values of real-time PCR assays ^34^. The cycle threshold refers to the number of thermal cycles required for the fluorescence signal to cross the assay’s diagnostic intensity threshold and therefore should be inversely proportional to the amount of target nucleic acid present in the specimen tested ^35^. However, whereas during the pre-ART era HIV-infected infants usually had high-level viremia, making a diagnosis straightforward ^36^, there has been a significant decrease in pre-treatment viral load associated with PMTCT practices ^37^. Hence, HIV-infected infants increasingly present for testing with low-level viremia and may even be aviremic ^35^. This in turn can result in a high number of HIV-infected infants who test indeterminate; South Africa reported around 3000 indeterminate results per annum between 2013 and 2015 ^38^, and birth cohorts found that half of infants with an indeterminate result were HIV-infected ^29, 39^. As both HIV PCR and viral load tests are recommended for the diagnosis of HIV in infants ^11^ and global demand for HIV viral load assays is far greater than for PCR assays, the temptation to simplify logistics and reduce costs of virological testing by using only viral load tests needs to be tempered with the likelihood of an increasing number of aviremic HIV-infected infants from exposure to antiretroviral drugs.

Similar to the challenges of confirming HIV infection status among infants who screen positive, the management of infants who test indeterminate can be complicated by antiretroviral drug pressure. Repeat indeterminate and false-negative PCR results can arise, straining clinical and laboratory services and the trust of caregivers. Not surprisingly, indeterminate results are associated with a high loss to follow-up rate and delays in making a definitive diagnosis and ART initiation in those who are HIV-infected ^38– 40^. As a means of reducing the indeterminacy rate without compromising accuracy, alternative verification methods have been proposed. Although data are limited, specimens that yield a reproducible HIV-detected result on repeat testing, even at high cycle threshold values, predict a positive HIV status. This has led to recommendations that all specimens that yield an EID non-negative result be repeat-tested on the same assay and that reproducible HIV-detected EID results be verified as positive whilst irreproducible HIV-detected results be verified as indeterminate ^33, 41^. Furthermore, infants who repeatedly test indeterminate should be managed as HIV-positive.

## Elimination of mother-to-child transmission and the future of early infant diagnosis

Prompted by the effectiveness of antiretroviral prophylaxis, the WHO has defined criteria for validating elimination of mother-to-child transmission (EMTCT). Although there is evidence that zero intrauterine and intrapartum infections can be achieved among women living with HIV who remain virologically suppressed throughout pregnancy ^42^, zero transmission is an unrealistic target for PMTCT programs. Rather, impact targets for EMTCT have been defined as a less than 5% transmission rate among breastfeeding populations (<2% among non-breastfeeding) and an HIV case rate of less than 50 infections per 100,000 total live births ^43^. Yet although some priority countries achieve (or nearly achieve) a 5% mother-to-child transmission rate, countries with a high maternal HIV prevalence are unlikely to achieve this case rate target. South Africa, for example, has struggled to effectively address a high HIV incidence among young women, and the antenatal HIV prevalence has remained around 30% for over a decade ^44, 45^. Consequently, a 1% mother-to-child transmission rate in South Africa, which has about a million live births per annum, equates to a case rate five times above the elimination target. Hence, despite a low mother-to-child transmission rate, South Africa can expect a considerable absolute burden of HIV-infected infants for the near future, emphasizing the continued relevance of accurate and timely EID services.

Future PMTCT developments are likely to further impact on the accuracy of EID assays. Dolutegravir (DTG), a potent antiretroviral capable of rapid viral suppression, is poised for widespread introduction into maternal ART programs. Because DTG is readily transferred to infants, both transplacentally ** and via breastmilk, further reductions in mother-to-child transmission are anticipated. This not only is expected to impact on the positive predictive value of EID tests but also may reduce the diagnostic sensitivity of nucleic acid–based assays. Surveillance during the first 24 months of life to monitor the effect of DTG on detection of HIV infection is essential to understand whether infant diagnostic guidelines require a total overhaul. For example, definitive diagnosis may not be possible until sometime after weaning. In adults, smaller HIV reservoirs are associated with a longer time to viral rebound ^46^. This is likely to apply to infants too where the combination of maternal ART and early ART for prophylaxis and treatment is associated with rapid decline of HIV-infected cells to low or undetectable levels ^47^. Hence, HIV-infected infants who achieve rapid and continuous viral suppression soon after birth could experience a prolonged period of time to viral rebound after weaning. Although data from the Children with HIV Early Antiretroviral Therapy (CHER) study suggest that almost all HIV-infected infants will experience virological rebound within 8 months of treatment cessation ^48^, these findings predate WHO Option B+ PMTCT practices and may not apply to intrauterine infected infants exposed to suppressive ART soon after infection. Therefore, documenting the age at which the majority of transmissions occur, and the timing of viral rebound among infected infants suppressed due to maternal and prophylactic antiretroviral drug exposure, will be necessary for future rational guideline development.

The distinction between intrauterine, intrapartum, and postnatal transmission has always been fairly crude but the boundaries have been further blurred by ART exposure as evidenced by case descriptions of infants who test HIV PCR indeterminate at birth followed by negative results and subsequent viral rebound after weaning, suggesting either suppressed intrauterine infection or postnatal transmission ^40^. Consequently, prognostic indicators for rapid disease progression in infants, other than intrauterine infection or high viral loads (which may be masked by ART), will be required. With increasing postnatal versus perinatal transmission, consideration should be given to the probability that an unknown proportion of these cases represent previously suppressed perinatal infections.

## Summary

The introduction of a single HIV PCR test for all HIV-exposed infants in low- and middle-income countries in the early 2000s represented significant progress for pediatric HIV outcomes. Today, EID algorithms require at least three HIV PCR tests for every exposed infant as well as testing at 18 months and after weaning. With high maternal HIV seroprevalence rates, the costs of the EID program are escalating, yielding fewer HIV-infected infants and more complex diagnostic dilemmas. In the quest for EMTCT, striving for virological suppression of all women living with HIV of childbearing potential will compound the complexities of an early definitive diagnosis of HIV. Consequently, false-negative and false-positive HIV PCR results are increasingly likely despite highly accurate diagnostic assays. The introduction of ART with even higher viral suppressive properties will require a complete rethink of efficient diagnostic algorithms, paired with more sensitive assays, for HIV diagnosis of infants and children in the future.
